# In Silico Identification of Novel Inhibitors Targeting the Homodimeric Interface of Superoxide Dismutase from the Dental Pathogen *Streptococcus mutans*

**DOI:** 10.3390/antiox11040785

**Published:** 2022-04-15

**Authors:** Carmen Cerchia, Emanuela Roscetto, Rosarita Nasso, Maria Rosaria Catania, Emmanuele De Vendittis, Antonio Lavecchia, Mariorosario Masullo, Rosario Rullo

**Affiliations:** 1 “Drug Discovery” Laboratory, Department of Pharmacy, University of Naples Federico II, 80131 Naples, Italy; carmen.cerchia@unina.it (C.C.); antonio.lavecchia@unina.it (A.L.); 2 Department of Molecular Medicine and Medical Biotechnology, University of Naples Federico II, 80131 Naples, Italy; emanuela.roscetto@unina.it (E.R.); mariarosaria.catania@unina.it (M.R.C.); devendit@unina.it (E.D.V.); 3 Department of Human Movement Sciences and Wellness, University of Naples “Parthenope”, 80133 Naples, Italy; rosarita.nasso@collaboratore.uniparthenope.it; 4 CEINGE, Biotecnologie Avanzate S.C.a R.L., 80145 Naples, Italy; 5 Institute for the Animal Production Systems in the Mediterranean Environment, 80147 Naples, Italy

**Keywords:** Superoxide dismutase, *Streptococcus mutans*, virtual screening, novel inhibitors, antibiofilm activity, dental plaque

## Abstract

The microaerophile *Streptococcus mutans*, the main microaerophile responsible for the development of dental plaque, has a single cambialistic superoxide dismutase (*Sm*SOD) for its protection against reactive oxygen species. In order to discover novel inhibitors of *Sm*SOD, possibly interfering with the biofilm formation by this pathogen, a virtual screening study was realised using the available 3D-structure of *Sm*SOD. Among the selected molecules, compound **ALS-31** was capable of inhibiting *Sm*SOD with an IC_50_ value of 159 µM. Its inhibition power was affected by the Fe/Mn ratio in the active site of *Sm*SOD. Furthermore, **ALS-31** also inhibited the activity of other SODs. Gel-filtration of *Sm*SOD in the presence of **ALS-31** showed that the compound provoked the dissociation of the *Sm*SOD homodimer in two monomers, thus compromising the catalytic activity of the enzyme. A docking model, showing the binding mode of **ALS-31** at the dimer interface of *Sm*SOD, is presented. Cell viability of the fibroblast cell line BJ5-ta was not affected up to 100 µM **ALS-31**. A preliminary lead optimization program allowed the identification of one derivative, **ALS-31-9**, endowed with a 2.5-fold improved inhibition power. Interestingly, below this concentration, planktonic growth and biofilm formation of *S. mutans* cultures were inhibited by **ALS-31**, and even more by its derivative, thus opening the perspective of future drug design studies to fight against dental caries.

## 1. Introduction

Superoxide dismutases (SODs) are key regulators of the redox homeostasis in organisms using the molecular oxygen for metabolism [1,2,3,4]. These metal-dependent enzymes, isolated from several sources, are usually grouped in three structurally unrelated families. For instance, mammals have a Cu/Zn-SOD (SOD1) in both cytosol and mitochondrial intermembrane space, a Mn–SOD (SOD2) in the mitochondrial matrix, and an extracellular Cu/Zn-SOD (SOD3); on the other hand, many eubacteria and archaea usually contain a Fe-SOD and/or a Mn–SOD, both belonging to the same group of SOD2 [5,6,7,8]. This simple classification is enriched for the occurrence of structurally unrelated Ni-SOD in *Streptomyces* [9], redundant Cu/Zn-SOD or Fe/Zn-SOD in some bacteria [10], single ‘cambialistic’ SOD in some microaerophiles [11,12,13,14,15,16,17]; indeed, the latter belongs to the SOD2 group and can function with either Fe or Mn in the active site. Despite their structural divergences, SODs keep a conserved catalytic mechanism, involving the dismutation of two superoxide anions into molecular oxygen and hydrogen peroxide [4,5,6,7,8]. During catalysis, the metal ion of the active site plays an important role, as it switches between two oxidation states. Another common feature to the different SODs is their organization in quaternary structures; indeed, depending on family and/or source, the monomer units of SODs are assembled to form homodimers, homotetramers or homohexamers. The minimum requirement for triggering activity is the homodimer [18]. Furthermore, SODs are usually endowed with a great resistance to physical and chemical agents, a property deriving from their intrinsically compact structure [19].

The subcellular compartmentalization of three different SODs in mammals is linked to a specific and pivotal role played by each enzyme. Indeed, an alteration of SOD functions may have dramatic consequences: for instance, the pathogenesis of amyotrophic lateral sclerosis or Alzheimer’s disease is strongly linked to a genetic alteration of SOD1 [2,20]; a defective SOD2 is incompatible with life, as indicated by the knocked-out mice model [21]; and cardiovascular diseases are probably linked to point mutations of SOD3 [22]. Furthermore, the abnormal production of superoxide anions in cancer cells renders them more dependent on SOD2 activity and modulators, with respect to normal cells [23,24]. All these features indicate that the usage of SOD as a target for the design of small molecules capable of altering enzyme functions should take into careful consideration the different role of the various SODs in redox homeostasis. In other words, a common SOD inhibitor would be inadequate, because of the relevance of the different SODs for cell survival. The main goal would be the identification of highly specific compounds capable of regulating a crucial function played by a SOD enzyme during the insurgence/progression of a disease.

Dental caries is one of the most common diseases, also frequently occurring among civilized populations. A definitive strategy for an efficient treatment of this multifactorial process is still missing, even though the gram-positive bacterium *Streptococcus mutans* has been recognized as the main microaerophile responsible for the development of caries [25,26,27]. This pathogen is a microaerophile that has been colonizing the oral cavity since the appearance of the first deciduous teeth; together with other microorganisms, it nestles into thin biofilms covering the tooth, in turn giving rise to bacterial plaque. In spite of its fermentative metabolism, *S. mutans* is considered a facultative aerobic microorganism, as it tolerates the presence of oxygen [28,29]. For its protection against reactive oxygen species, *S. mutans* has the single ‘cambialistic’ SOD (*Sm*SOD) [30,31,32]. This enzyme exhibits a higher affinity for Fe incorporation with respect to Mn, but its Mn-bound form is 56-fold more active than the Fe-bound one [11]. Furthermore, *Sm*SOD sensitivity to physiological inactivators is regulated by the type of metal. The metal-dependent modulation of SOD functions is particularly evident in *S. mutans*, because in the non-pathogenic homologue *S. thermophiles*, the corresponding ‘cambialistic’ *St*SOD is much less regulated [12]. The characterization of *Sm*SOD and *St*SOD included the determination of their 3D-structures [13]; both enzymes belong to the Mn-like subgroup of ‘cambialistic’ SODs, although with great differences in regulation of their properties depending on bound metal. These data are particularly interesting, as they could explain why a high concentration of Mn in drinking water and diet correlates with the increasing incidence of dental caries [33,34,35].

**Figure 1:**
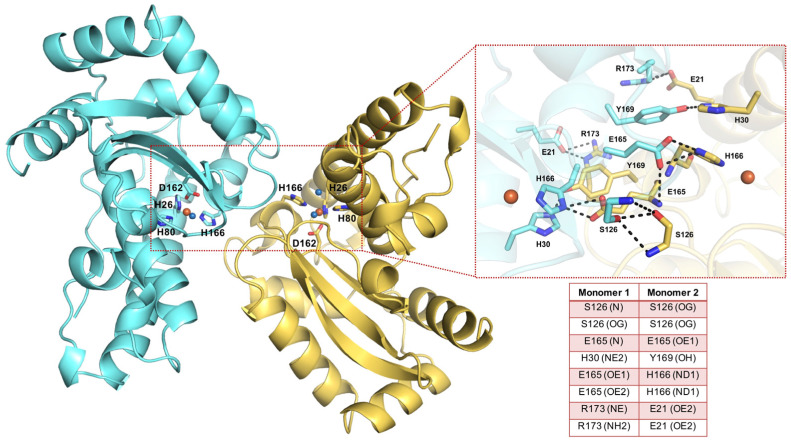
Overview of *Sm*SOD architecture and residues at the dimer interface. The X-ray structure of *Sm*SOD (PDB 4YIP) is shown as a ribbon model, with the two monomers coloured in aquamarine and yellow. The active site residues (H26, H80, H166, D162) are shown as sticks; the metal ion and the coordinating water molecules are shown as orange and blue spheres, respectively. On the right side, a zoom-in of the residues composing the dimer interface in which H-bonds are depicted as black dashed lines and listed in the inserted table.

Both cambialistic SODs adopt the topology of Fe/Mn–SODs, consisting of a symmetric dimer formed by two identical monomers. The strictly conserved active site of Mn–SODs in *Sm*SOD structure lies on the interface between these two monomers and comprises three histidine residues (H26, H80 and H166), one aspartic acid (D162) and a water molecule that coordinates the metal ion in a trigonal–bipyramidal geometry (Figure 1). A peculiar feature of the *Sm*SOD structure is the presence, in one monomer, of an additional water molecule functioning as a sixth ligand and changing the metal coordination geometry to a distorted octahedron. Moreover, the catalytic activity of SOD enzymes heavily relies on their quaternary structure, and the dissociation of dimers into monomers results in the loss of enzymatic activity [13]. In the case of *Sm*SOD, the dimer interface is stabilized by a number of hydrophobic and polar interactions, these latter detailed in the zoom-in of Figure 1. Given the binary symmetry of the *Sm*SOD dimer, the interactions between the two chains are perfectly symmetric.

**Figure 2:**
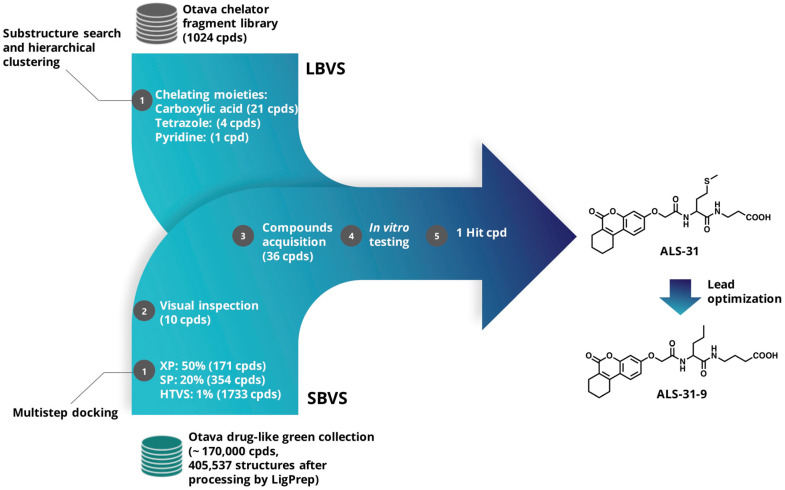
Flow chart of Ligand-based (LBVS) and Structure-based (SBVS) virtual screening approaches.

In this work, a virtual screening study was undertaken with the aim of discovering small molecules acting as possible inhibitors of *Sm*SOD, and possibly interfering with the biofilm formation by this dental pathogen. Due to the key role of the metal ion in SOD catalytic mechanism, the interaction of the active site metal ion with a small molecule inhibitor could be an efficient way of deactivating the antioxidant effect of this enzyme. In addition, a deeper examination of the available 3D-structure of *Sm*SOD [13] highlighted the possibility of targeting the dimer interface and disrupting the quaternary structure, whose integrity is critical for the enzymatic activity. Therefore, two main virtual screening (VS) approaches were pursued, namely Ligand-based (LBVS) [36,37] and Structure-based (SBVS) [38], schematized in Figure 2 and detailed below. Among the molecules selected by the VS process, compound **ALS-31** showed the ability to inhibit the activity of *Sm*SOD and that of other typical SODs from bacterial and eukaryal sources. A preliminary lead optimization program on this novel compound allowed the identification of one derivative, **ALS-31-9**, possessing an improved inhibition power compared to the lead compound. **ALS-31**, and more efficiently, its derivative **ALS-31-9**, are able to inhibit both the planktonic growth and the biofilm formation of *S. mutans*.

## 2. Materials and Methods

### 2.1. Materials and Reagents

Compounds **ALS-1→36** and **ALS-31-1→9** were purchased from Otava (http://www.otavachemicals.com/, accessed on 28 September 2015). According to the suppliers’ information, the compounds were at least 90% pure and stock solutions were prepared in dimethylsulfoxide (DMSO) at 20 mM concentration. Dulbecco’s modified Eagle’s medium (DMEM), Medium 199 (cat. BE12-117F), foetal bovine serum (FBS, cat. BP12-725F), L-glutamine (cat. BE17-605E), penicillin G-streptomycin (cat. 17-602F) and trypsin (cat. BE17-161E) were purchased from Lonza (Milano, Italy). Ampicillin (cat. A1593), kanamycin (cat. K1377), isopropyl-β-thiogalactopiranoside (IPTG, cat. I6758), xanthine (cat. X0626), xanthine oxidase (cat. X1875) and cytochrome *c* (cat. C2506) were from Sigma–Aldrich (St. Louis, MO, USA). The chromatographic medium Ni-NTA agarose (cat. 30210) was from Qiagen (Milano, Italy). Aquacide IIA (cat. 9004-32-4) was from Calbiochem-Merk (Darmstadt, Germany). HPLC-grade solvents for mass spectrometry were obtained from Carlo Erba. A protease inhibitor cocktail (cat. 04693116001) was obtained from Roche Diagnostics (Indianapolis, IN, USA). Rabbit polyclonal antibody against human SOD2 (cat. 06-984) was purchased from Millipore (Milano, Italy). Rabbit monoclonal antibody against GAPDH (cat. 2118s) was purchased from Cell Signaling Technology (Danvers, MA, USA) and HRP conjugated secondary antibody (cat. #170-6515) was purchased from Santa Cruz Biotechnology (Heidelberg, Germany). All other reagents and solvents of high analytical grade were from Sigma–Aldrich (St. Louis, MO, USA).

### 2.2. Computational Methods

#### 2.2.1. Ligand Database Preparation and Virtual Screening Protocol

For LBVS, the chelator fragment library [39] was obtained upon request from Otava and processed using the RDKit nodes implemented in KNIME (Konstanz Information Miner, version 3.3.2) [40]. This focused library comprises ligands with chelating groups with a high propensity to bind metal ions. The compounds’ physicochemical properties accomplish an expanded Lipinski’s “Rule of Three”. The library (containing 1024 ligands at the time of the study) was exposed to a substructure search, using as query the following SMARTS notations: [#6]-[#6](=[#8])-[#8]-[#1], c1nnnn1, and [#6]-c1ccncc1, corresponding to carboxylic acid moiety, tetrazole and pyridine groups, respectively. The substructure searches yielded 319 compounds bearing the carboxylic acid moiety, 11 compounds possessing the tetrazole group and 63 bearing the pyridine group. Then, we applied an agglomerative hierarchical clustering based on the Morgan circular fingerprints (radius 2), with a Tanimoto distance threshold of 0.5. This procedure allowed us to more efficiently prioritize 21 compounds with the carboxylate moiety, 4 compounds bearing the tetrazole and 1 compound with the pyridine group, taking also into account the compounds’ availability. Hence, a total of 26 compounds (**ALS-1→17**, **ALS-19→20** and **ALS-22→28** in Table S1) were obtained from this first library for biochemical evaluation.

For SBVS, the “drug-like green collection” (~170,000 compounds) was retrieved from the Otava website; the tridimensional structures of the compounds were generated and prepared using LigPrep module (LigPrep, Schrödinger, LLC, New York, NY, USA, 2021), predicting all tautomeric and protonation states at pH 7 ± 2. This yielded a total of 405,537 structures. The X-ray coordinates of *Sm*SOD (PDB 4YIP) [13] were used as the structural template for SBVS and processed with the “Protein Preparation Wizard” workflow [41]. Water molecules were removed, with the exception of those completing the metal center coordination sphere, and the appropriate bond orders as well as charges and atom types were assigned, and the hydrogen atoms were added. The H-bond network was optimized by exhaustive sampling of rotamers, tautomers and protonation states of titratable amino acids at neutral pH. Finally, the protein structure was optimized by running a restrained minimization using the Impref module with the OPLS_2005 force field, by imposing a 0.3 Å RMSD limit from the initial coordinates as constraint. Then, a docking grid was generated using the “Receptor Grid Generation” module of Maestro, enclosing a box centered on E165, which lies in the interface between the two *Sm*SOD monomers, with an inner box size of 10 × 10 × 10 Å and an outer box of 30 × 30 × 30 Å. A scaling factor of 0.8 was set for Van der Waals radii of receptor atoms. Ligand sampling was allowed to be flexible. The multistep docking strategy implemented is also summarized in Figure 2. Briefly, the top-ranked compounds passing Glide HTVS stage (1733 compounds) were docked flexibly in a stepwise manner with Glide Standard Precision (SP) and Extra Precision (XP) [42,43,44]. For these two steps, we employed default parameters with the same receptor grids used in Glide HTVS stage. Out of the final 171 compounds identified from the SBVS approach, 10 (**ALS-18**, **ALS-21** and **ALS-29→36** in Table S1) were selected for experimental testing on the basis of visual inspection and compounds’ availability. The selected compounds were further checked for known classes of pan-assay interference compounds (PAINS) by using Faf-Drugs4 [45]. None of the compounds were found as potential PAINS.

#### 2.2.2. Refinement of the Lead Compound **ALS-31**

In order to find structurally similar compounds to **ALS-31**, a 2D similarity-based search was used to interrogate the entire Otava drug-like green collection. Again, the RDKit nodes in KNIME and Morgan circular fingerprints (radius 2) were employed in this step. The top 100 most similar compounds, ranked by Tanimoto similarity, were visually analyzed; finally, on the basis of compounds’ availability, we selected and ordered 12 molecules (named **ALS-31-1→12**) for biological testing.

### 2.3. Biochemical and Biological Methods

#### 2.3.1. SOD Samples and Enzymatic Assays

Purified recombinant forms of *Sm*SOD and *St*SOD were obtained through a heterologous expression system constituted by the *Escherichia coli* BL21(DE3) strain transformed with appropriate expression vectors, v*Sm*SOD [11] or v*St*SOD [12]. These pET-22b(+)- or pET-28b(+)-derived plasmids led to the production of recombinant forms of *Sm*SOD or *St*SOD, respectively fused to a His-tail, useful for their one-step purification by affinity chromatography, as previously described [11,12]. The improvement of Mn uptake by *Sm*SOD was realised as previously described [11,12,13]. The resulting metal (Fe and Mn) content in the cambialistic enzyme was determined by graphite furnace atomic absorption spectrometry with a Shimadzu ACS-6100 auto sampler [46]. Fe-SOD from *Escherichia coli* (cat. SRP6107) and the Cu/Zn-SOD from bovine erythrocytes (cat. 574594) were purchased from Sigma–Aldrich. A recombinant form of the eukaryal Mn–SOD from rat mitochondria was prepared as previously described [19]. Protein concentration was determined by the Bradford method, using bovine serum albumin as standard [47]. SOD activity was measured at 25 °C in 100 mM potassium phosphate buffer, pH 7.8 and 0.1 mM Na-EDTA by the inhibition of cytochrome *c* reduction caused by superoxide anions generated with the xanthine/xanthine oxidase method [48,49]. One unit of SOD activity was defined as the amount of enzyme that caused 50% inhibition of cytochrome *c* reduction.

#### 2.3.2. Size Exclusion Chromatography

The oligomerization status of *Sm*SOD was analyzed by gel-filtration on a Superdex^TM^ 75 10/300 GL column (GE Healthcare) connected to an FPLC^TM^ system. The column was equilibrated and eluted at 0.5 mL/min at room temperature (20–24 °C) with a 20 mM Tris•HCl buffer, pH 7.8, containing 150 mM KCl and 1% (*v*/*v*) DMSO. However, where indicated, the equilibration and elution buffer also contained 200 µM inhibitor. The protein molecular mass standards used for calibration of the size exclusion chromatography were bovine serum albumin (68 kDa), egg albumin (46 kDa), carbonic anhydrase (30 kDa) and cytochrome *c* (12.4 kDa).

#### 2.3.3. Cell Line and Culture Conditions

The cell line BJ-5ta, a human skin fibroblast immortalised with the human telomerase reverse transcriptase (Cell Culture Facility of CEINGE, Naples, Italy), was cultured in a 4:1 mixture of DMEM and Medium 199 supplemented with 4 mM L-glutamine, 4.5 g/L glucose, 1.5 g/L sodium bicarbonate, 10% FBS, 100 IU/mL penicillin G and 100 mg/mL streptomycin in a humidified incubator at 37 °C under 5% CO_2_ atmosphere. Cells were split and seeded every 3 days and used during their exponential phase of growth. Cell treatments were usually carried out after 24 h from plating. To obtain total protein extracts from BJ5-ta, cells were seeded into six-well plates (3 × 10^5^ cells/plate) for 24 h at 37 °C and then treated with **ALS-31** or 0.5% (*v*/*v*) DMSO. After treatment, cells were harvested, washed with PBS and then lysed in ice-cold modified radio immunoprecipitation assay (RIPA) buffer (50 mM Tris•HCl, pH 7.4, 150 mM NaCl, 1% Nonidet P-40, 0.25% sodium deoxycholate, 1 mM Na_3_VO_4_ and 1 mM NaF), supplemented with protease inhibitors and incubated for 30 min on ice. The supernatant, obtained after centrifugation at 13,200 g for 30 min at 4 °C, constituted the total protein extract.

A colony-forming assay was performed as previously described, with some modifications [50]. Briefly, BJ-5ta cells were seeded in duplicate in six-well plates at a density of 4 × 10^2^ cells per well. After 2/3 days, cells were treated with 0.5% (*v*/*v*) DMSO or different **ALS-31** concentrations and incubated for additional 10 days at 37 °C. Then, colonies were stained with 1% (*w*/*v*) crystal violet in 50% (*v*/*v*) ethanol for 1 h at room temperature. Cells were photographed with a digital camera (Canon PowerShot G9); the number of colonies (≥50 cells per colony) was counted using ImageJ 1.42q software.

#### 2.3.4. Western Blotting Analysis

Equal amounts of total protein extracts obtained from BJ5-ta cells were used for Western blotting analysis. Briefly, protein samples were dissolved in SDS-reducing loading buffer, run on sodium dodecylsulfate polyacrylamide gel electrophoresis (SDS/PAGE) and then transferred to Immobilon P membrane (Millipore, Saint Louis, MO, USA). The filter was incubated with the rabbit polyclonal antibody against human SOD2 at 4 °C overnight and then with the HRP conjugated secondary antibody at room temperature for 1 h. Membranes were then analysed by an enhanced chemiluminescence reaction using WesternBright ECL (cat. NC0930892, Advansta, San Jose, CA, USA) according to manufacturer’s instructions; signals were visualised by autoradiography.

### 2.4. Microbiological Assays

The bacterial strain used for antimicrobial assays was *Streptococcus mutans* ATCC-700610. The strain was stored in 15% (*v*/*v*) glycerol stocks at −80 °C. Before each experiment, cells were subcultured from the stocks into Trypticase Soy Agar (TSA, cat. PA-254051.06) with 5% Sheep Blood (Becton Dickinson, Sparks, NV, USA) plates at 37 °C in 5% CO_2_ atmosphere for 48 h.

The antibacterial activity of compounds **ALS-31** or **ALS-31-9** was assayed in *S. mutans* cultures by a standard broth micro-dilution method in 96-well polystyrene plates using Brain-Heart Infusion broth (BHI, cat. 255003, Becton Dickinson, Sparks, NV, USA). One hundred microliters of bacterial suspension adjusted to approximately 1.5 × 10^6^ CFU/mL were incubated with 100 µL of serial dilutions of the compounds dissolved in final 1.0% (*v*/*v*) DMSO as a vehicle. The plates were incubated at 37 °C in 5% CO_2_ atmosphere for 24 h under shaking (300 rpm). The medium turbidity was measured by a spectrophotometer at 595 nm (Bio-Rad Laboratories Inc., Hercules, CA, USA). Compound-free wells in 1.0% (*v*/*v*) DMSO were used as controls (100% planktonic growth), and antibacterial activity was expressed as a percentage of residual growth. Chlorhexidine acetate (CHX, cat. 51094, Sigma-Aldrich, St. Louis, MO, USA) solution (1 mg/mL) was prepared and included as control for the assay of *S. mutans*’ susceptibility (MIC referred to [51]). Each compound was tested in triplicate; each experiment was performed twice.

The crystal violet (CV) staining method was used to measure biofilm biomass formed in flat-bottomed 96-well microplates in the presence of **ALS-31** or **ALS-31-9**, as described by Stepanović et al., with some modifications [52]. A bacterial suspension of approximately 1.5 × 10^6^ CFU/mL in BHI supplemented with 1% (*w*/*v*) glucose was prepared, and 100-µL aliquots of this suspension were incubated with 100 µL of serial dilutions of each compound. The microplate was incubated at 37 °C for 24 h, after which the non-adherent cells were gently aspirated and the wells gently rinsed with PBS (Sigma–Aldrich). The adhered biofilm was dried at 60 °C for 30 min and subsequently stained with 0.1% (*w*/*v*) crystal violet solution for 30 min. After washing with PBS and solubilization with absolute ethanol to release the dye from the biofilm, the plate was read at 570 nm in a spectrophotometer. The absorbance recorded was related to the amount of biofilm produced. The reduction in the mass of the biofilm was expressed as a percentage by using the formula [(Ac-At)/Ac] × 100, where Ac is the OD_570_ for the control wells and At is the OD_570_ in the presence of the tested compound.

### 2.5. Statistical Analysis

Data are reported as the mean ± standard error (SE). The statistical significance was evaluated with the Student’s t-test and the significance was accepted when *p* < 0.05.

## 3. Results and Discussion

### 3.1. Identification of Small Molecule Inhibitors Targeting the SmSOD by LBVS and SBVS

In the search of novel inhibitors of *Sm*SOD, which is an essential enzyme for dental pathogen survival, we established two main VS approaches, LBVS and SBVS, schematized in Figure 2. Since the metal center has a critical role in *Sm*SOD catalysis, alterations in the active site induced by metal chelation or modifications in the coordination geometry could effectively deactivate its antioxidant action and, therefore, influence both the growth and survival of the pathogen. Thus, we built a SMARTS-based query to screen the Otava chelator fragment library (see Materials and Methods for details) by means of RDKit nodes implemented in KNIME. We prioritized ligands furnished with functional groups, which are well-known to potentially act as metal chelators, such as carboxylic acid, tetrazole and pyridine moieties [53]. Iron and other metal ions are positively charged and are often bound to ligands containing electron donor atoms such as O and N, capable of donating a pair of electrons for the formation of a coordinated bond with the metal. In this regard, ligands functionalized with sp^2^ and sp^3^ (NH_2_) nitrogen atoms, such as heterocyclic units (pyridine or amino groups), have been previously reported as SOD inhibitors of different pathogens [54,55]. The substructure search and clustering procedures allowed the easy selection and acquisition of 26 compounds for biological testing (Table S1).

On the other hand, the availability of the X-ray structure of *Sm*SOD (PDB 4YIP) [13] prompted us to set up a SBVS protocol to identify new chemical scaffolds able to disrupt the enzyme dimer interface. The 3D structure of the *Sm*SOD shows that residues S126, E165, H30, Y169, H166, R173 and E21 are mainly involved in dimer assembly (Figure 1) and thus constitute possible ‘hot spots’. In this light, a multi-step SBVS of about 170,000 drug-like compounds obtained from Otava was conducted using the Glide software. The *Sm*SOD dimer interface was the region targeted for docking; the resulting 171 top-ranked compounds were further analyzed and, finally, 10 compounds were selected. In total, 36 compounds were purchased (listed in Table S1).

### 3.2. Biochemical Effects Exerted by the Novel Compounds on SmSOD

#### 3.2.1. Inhibition of the Activity of *Sm*SOD

The activity of *Sm*SOD was considered as a convenient tool to study the effects of the compounds identified by virtual screening on the properties of this enzyme. Measurements of SOD activity are usually performed through an indirect assay method that uses other proteins, i.e., the enzyme xanthine oxidase and cytochrome *c*. To this aim, an evaluation was made of whether any among thirty-six compounds added at 50 µM or 200 µM interfered with this indirect SOD assay. Indeed, in the absence of *Sm*SOD, the rate of cytochrome *c* reduction caused by superoxide anions arising from the xanthine oxidase activity was affected by four compounds (**ALS-1**, **-8**, **-15** and **-28**; not shown). Therefore, these molecules could not be tested as possible effectors of *Sm*SOD activity when using the indirect SOD assay. The effect of the remaining thirty-two molecules (**ALS-2→7**, **ALS-9→14**, **ALS-16→27** and **ALS-29→36**) on *Sm*SOD activity was evaluated, and the residual activity measured in the presence of 20, 50 or 200 µM concentration of any compound is shown in Table 1. Most of them were apparently ineffective, as they provoked a roughly dose-independent effect on *Sm*SOD activity, whereas **ALS-31** seemed to cause a dose-dependent reduction in the activity.

**Figure 3:**
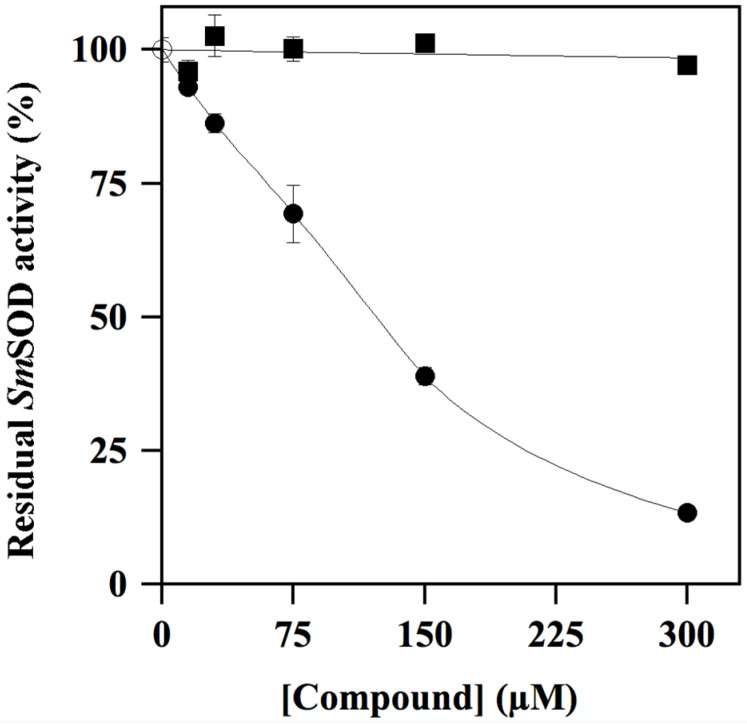
Effect of **ALS-31** or **ALS-19** on the activity of *Sm*SOD. The SOD activity was measured in triplicate, as indicated in the Materials and Methods, and reported as the mean ± SE. Values of residual activity were expressed as a percentage of the activity measured in the absence (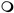) or in the presence of the indicated concentration of **ALS-31** (●) or **ALS-19** (∎).

To get an insight on this effect, inhibition profiles up to 300 µM were realised for two compounds, namely the putative inhibitor **ALS-31** and an apparently ineffective molecule, such as **ALS-19**. The results of a representative experiment (Figure 3) clearly indicate that only **ALS-31** caused an evident dose-dependent inhibition of *Sm*SOD activity. The calculated value of the **ALS-31** concentration, leading to 50% inhibition of *Sm*SOD activity (IC_50_), was 159 ± 19 µM.

The biochemical properties of the cambialistic *Sm*SOD are greatly regulated by its metal content, i.e., Fe and Mn, as demonstrated in samples containing a different Fe/Mn ratio in the binding pocket of this enzyme [11,12]. In particular, after an improvement in the Mn content at the expense of Fe, the specific activity of *Sm*SOD significantly increased and, interestingly, its sensibility to typical SOD inhibitors was also altered. This latter observation prompted an investigation into the sensibility to inhibition by **ALS-31**, when the basal Mn content of *Sm*SOD was 2.7-fold improved after a metal exchange reaction. A comparison of the inhibition profiles realised with the Mn-basal and Mn-enriched *Sm*SOD sample indicated that the IC_50_ value for **ALS-31** raised to 262 ± 11 µM in the Mn-enriched enzyme. This result indicates that resistance of *Sm*SOD to the inhibitor **ALS-31** was improved by the Mn content in the cambialistic enzyme.

#### 3.2.2. Effects of **ALS-31** on the Quaternary Structure of *Sm*SOD

**Figure 4:**
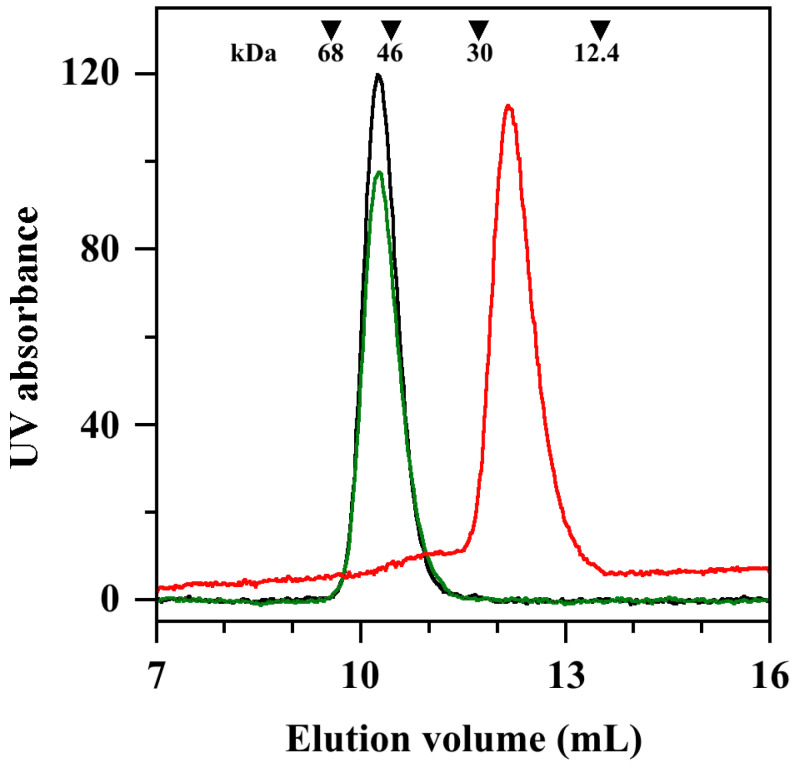
Effect of **ALS-31** on gel-filtration of *Sm*SOD. A protein sample of *Sm*SOD, 63 µM in 20 mM Tris•HCl buffer, pH 7.8, supplemented with 150 mM KCl and 1% (*v*/*v*) DMSO, was untreated or incubated at room temperature for 20 min with 200 µM **ALS-31**. The protein sample was then loaded on a Superdex 75 10/300 column and the elution profile was followed by a continuous absorbance monitoring at 280 nm. Untreated *Sm*SOD (black line); *Sm*SOD incubated with 200 µM **ALS-31** without (green line) or after the addition of 200 µM **ALS-31** in the elution buffer (red line). The elution volumes of bovine serum albumin (9.56 mL), egg albumin (10.44 mL), carbonic anhydrase (11.72 mL) and cytochrome *c* (13.51 mL), used as standard protein markers, are indicated by inverted triangles. Their position was unaffected by the addition of **ALS-31** in equilibration and elution buffer.

The conserved catalytic mechanism displayed by all SODs involves the sequential reduction and re-oxidation of the metal cation, with the concomitant oxidation and reduction of two superoxide anions. For this reason, the minimum requirement for triggering the enzymatic activity of SODs has been linked to the formation of the active form of the enzyme obtained through the assemblage of two monomers into a homodimer [18]. Indeed, *Sm*SOD is also organised as a homodimer [11]. As a possible mechanism for the inhibition properties exhibited by **ALS-31**, the disassemblage of the *Sm*SOD homodimer in the presence of the inhibitor was considered. To verify this hypothesis, a size exclusion chromatography was realised on a Superdex 75 10/300 column. The position of the single peak emerging from the elution profile of an untreated *Sm*SOD sample (Figure 4, black line) confirms that this enzyme is organised as a homodimer, because the calculated molecular mass of 51.8 kDa was close to the theoretical *M*_r_ 46.9 assigned to homodimer. The small discrepancy between experimental and theoretical value of molecular mass was already observed for both *Sm*SOD and *St*SOD [11,12] and was tentatively explained by the reversible formation of protein aggregates containing multiple homodimers at higher protein concentration. An almost identical elution was observed when the *Sm*SOD sample was previously incubated with 200 µM **ALS-31** before its loading (Figure 4, green line). On the other hand, if the equilibration and elution buffer of the column was enriched with 200 µM **ALS-31**, the position of *Sm*SOD in the elution pattern was profoundly modified (Figure 4, red line). Indeed, the major protein peak shifted towards higher elution volume, thus resulting in a calculated molecular mass of 23.2 kDa, a value essentially coincident with the predicted *M*_r_ 23.4 assigned to the *Sm*SOD monomer. This finding suggests that **ALS-31** provokes the dissociation of the *Sm*SOD homodimer in two monomers, a finding that compromises the catalytic activity of the enzyme. However, it is likely that the interaction between the enzyme and the inhibitor was reversible, because the dissociation into monomers was not observed when the inhibitor was absent in the eluting buffer. Interestingly, if **ALS-31** was replaced by an inactive compound, such as **ALS-9**, present at 200 µM in both protein sample and elution buffer, the *Sm*SOD eluted as a homodimer (not shown), thus indicating that this inactive compound did not affect the quaternary structure of the enzyme.

The putative reversible interaction between *Sm*SOD and **ALS-31** was further investigated through the behaviour of this enzyme in gel electrophoresis. Indeed, no change was observed in the electrophoretic mobility of *Sm*SOD when protein samples ranging from 7 to 51 µM, without or with their preincubation with 488 µM **ASL-31**, were analysed on electrophoresis gel run under not denaturing conditions (not shown). Therefore, the reversible and non-covalent interaction between *Sm*SOD and **ALS-31** allowed the disassemblage of the enzyme and consequently its inhibition, only when a proper concentration of the inhibitor remained in contact with the enzyme.

#### 3.2.3. Inhibition of Other SODs by **ALS-31**

**Figure 5:**
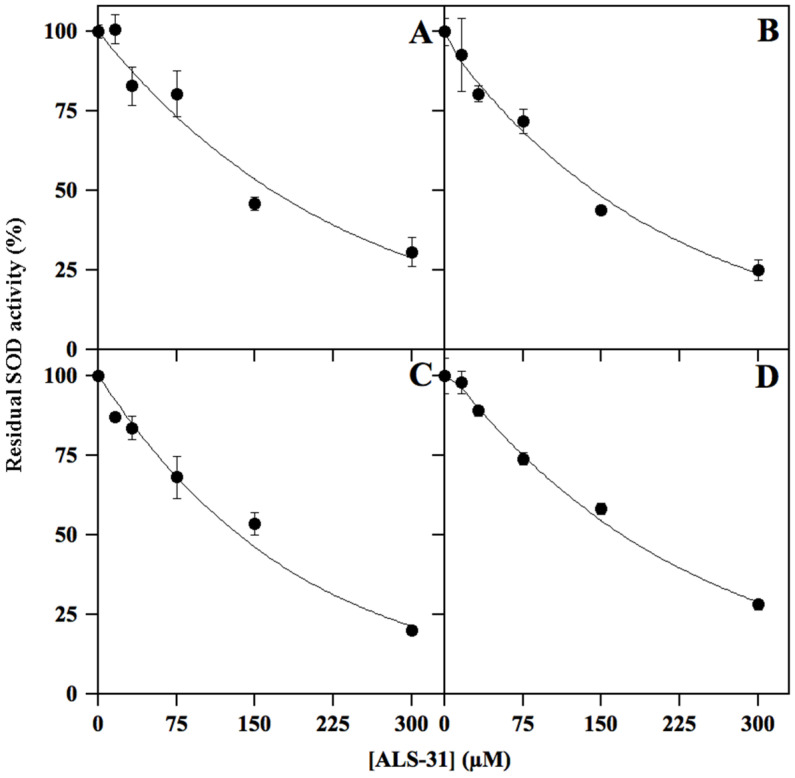
Representative effect of **ALS-31** on the activity of different SODs. The activity of *St*SOD (**A**), Fe-SOD from *Escherichia coli* (**B**), Mn–SOD from rat mitochondria (**C**) or Cu/ZnSOD from bovine erythrocytes (**D**) was measured and expressed (●) as indicated in Figure 3.

To evaluate the specificity of the inhibition caused by **ALS-31** on *Sm*SOD, we have considered other possible SOD targets from bacterial and eukaryal sources. To this aim, inhibition profiles by **ALS-31** were realised with the cambialistic *St*SOD (Figure 5A), the Fe-SOD from *E. coli* (Figure 5B), the Mn–SOD from rat mitochondria (Figure 5C) and the Cu/ZnSOD from bovine erythrocytes (Figure 5D). The data indicate that these typical bacterial and eukaryal SODs are also targets of the inhibition by **ALS-31**. When the IC_50_ calculated for these SODs was compared with the corresponding value obtained for *Sm*SOD (Table S2), a roughly similar inhibition power emerged among the different enzymes, a finding indicating that **ALS-31** could be ranked as a novel common inhibitor of SODs. Notably, the residues mainly involved in dimer assembly are well conserved among *St*SOD, Fe-SOD from *E. coli* and the Mn–SOD from rat mitochondria (Figure S1), whereas the Cu/ZnSOD from bovine erythrocytes, a member of the SOD1 subfamily, displays a remarkably different dimer architecture. Thus, a different mechanism might be involved in the inhibitory effect exerted by **ALS-31** on this enzyme.

### 3.3. Effect of **ALS-31** on Fibroblast Cell Line Viability

**Figure 6:**
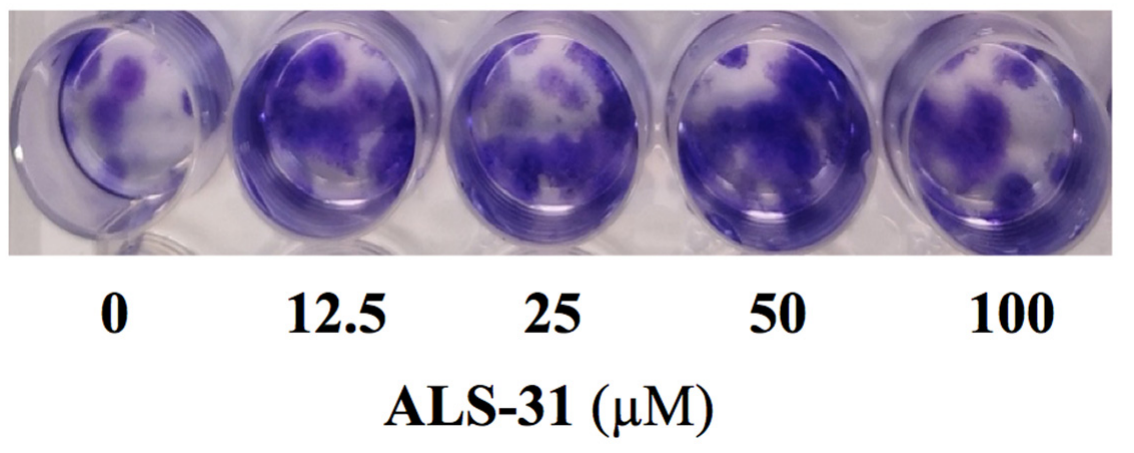
Effect of **ALS-31** on the colony formation of fibroblast cell line BJ-5ta. Cells were treated with vehicle alone or the indicated concentration of **ALS-31**. After 10 days treatment, plates were photographed and images of representative experiments are shown. Other details are as indicated in the Materials and Methods section.

The possible toxic effect of compound **ALS-31** was investigated using the nonmalignant human fibroblast cell line BJ-5ta, because it represents a ubiquitous cell type, even in mouth tissues. To evaluate a possible long-term effect on cell growth inhibition by this compound, a colony formation assay was realised by incubating fibroblasts in the presence of an increasing concentration of **ALS-31**, up to 100 µM. The incubation was prolonged up to 72 h and the data clearly indicate that this compound was not toxic for the growth of this normal cell line, even when added at 100 μM (Figure 6).

Furthermore, we have also evaluated whether **ALS-31** could affect the protein expression levels of the mitochondrial Mn–SOD. To this aim, total protein extracts were obtained from fibroblast cells treated as in the previous experiment and probed by Western blotting, using rabbit polyclonal antibodies against mitochondrial Mn–SOD. No significant differences existed among the protein levels of Mn–SOD resulting from treated or untreated cells (not shown).

### 3.4. Molecular Modelling and Lead Optimization Procedure

#### 3.4.1. Structural Basis for *Sm*SOD Inhibition by ALS-31

**Figure 7:**
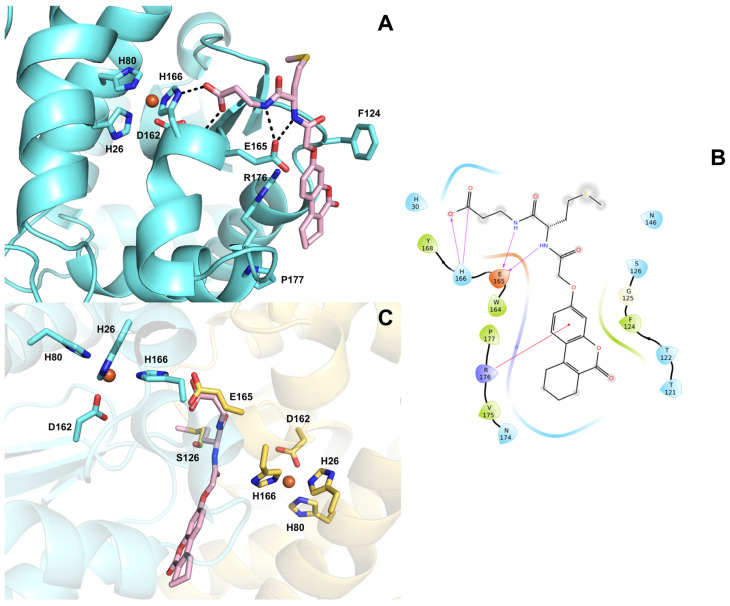
(**A**) Predicted binding mode of compound **ALS-31** (displayed as pink sticks) at the dimer interface of *Sm*SOD (PDB 4YIP). The amino acid side chains important for ligand binding are represented as sticks and labelled. The metal ion is represented as an orange sphere. Hydrogen bonds are shown as black dashed lines. (**B**) 2D ligand interaction diagram of compound **ALS-31**. Positively charged amino acids are represented with dark blue drops, negatively charged amino acids are represented with red drops, polar amino acids are represented with light blue drops and hydrophobic amino acids are represented with green drops. H-bonds are depicted with purple arrows. Straight red lines represent cation-π interactions. (**C**) Superposition of **ALS-31** docked pose with monomer A residues E165 and S126 (displayed as yellow sticks).

To provide an explanation at the molecular level for the inhibitory activity of **ALS-31** towards *Sm*SOD, a docking model is proposed, elucidating the putative interactions between this compound and the enzyme. **ALS-31** is hosted in a cleft at the dimer interface, establishing two strong H-bonds with both ND1 and N of H166 through its acidic group (Figure 7A,B). Moreover, the ligand amide nitrogen atoms engage two further H-bonds with E165. The coumarin moiety forms an additional cation-π interaction with the side chain of R176. It is worth noting that H166 and E165 are critical hot spots involved in dimer assembly; in addition, H166 is also part of the metal coordination sphere in the active site. The docking pose shows that the **ALS-31** acidic group is superimposable to E165 side chain on the other monomer, whereas the methyl(propyl)sulfane group nicely overlaps to S126 (Figure 7C). On the basis of this model, it can be hypothesized that compound **ALS-31** is able to compromise the enzyme dimeric structure by mimicking these key hot spots residues required for monomer association and might eventually also result in a perturbation of the metal coordination, thus impeding the enzyme catalytic activity. Interestingly, this ‘glutamate bridge’ (that is, the glutamate residue on one monomer H-bonded to the metal ligand histidine of the other monomer) is highly conserved in Fe- and Mn–SODs, implying an important role in protein structure. Indeed, point mutation of this residue to alanine has been reported to destabilize the dimer structure, with the protein mutant occurring as a mixture of dimer and monomer species [56].

#### 3.4.2. Lead Optimization of *Sm*SOD Inhibitor **ALS-31**

The most promising compound **ALS-31** was selected for similarity follow-up searches over the entire Otava drug-like green collection, in order to find structurally related molecules and possibly improve its inhibition properties. This search yielded twelve molecules, called **ALS-31-1→12**, whose structures are shown in Table 2. All novel analogues were assayed for their ability to inhibit the activity of *Sm*SOD when present in the assay at 50 or 100 µM (Table 2). Four out of twelve derivatives, namely **ALS-31-6**, **ALS-31-8**, **ALS-31-9** and **ALS-31-10**, seemed to cause a dose-dependent reduction in the *Sm*SOD activity, whereas the others were almost ineffective. Inhibition profiles were performed for these active molecules and the resulting IC_50_ values are reported in Table 2. All of them were capable of inhibiting the *Sm*SOD, although with a modulation of their inhibition properties. Interestingly, among the four analogues, **ALS-31-9** had an IC_50_ value (64 ± 15 µM) significantly lower than that obtained with the lead **ALS-31**. In particular, the optimized derivative **ALS-31-9** has a 2.5-fold improved inhibition power on *Sm*SOD. The structural differences between this derivative and the lead compound include the increased length, by one CH_2_, of the carboxylic arm of the derivative, as well as the elimination of the thioether function in the second arm of the compound, thus reducing its length. These modifications, resulting in a better functional interaction between *Sm*SOD and the optimized derivative, indicate the direction for further optimization.

### 3.5. Effect of **ALS-31** and **ALS-31-9** on Growth and Biofilm Formation of S. mutans

**Figure 8:**
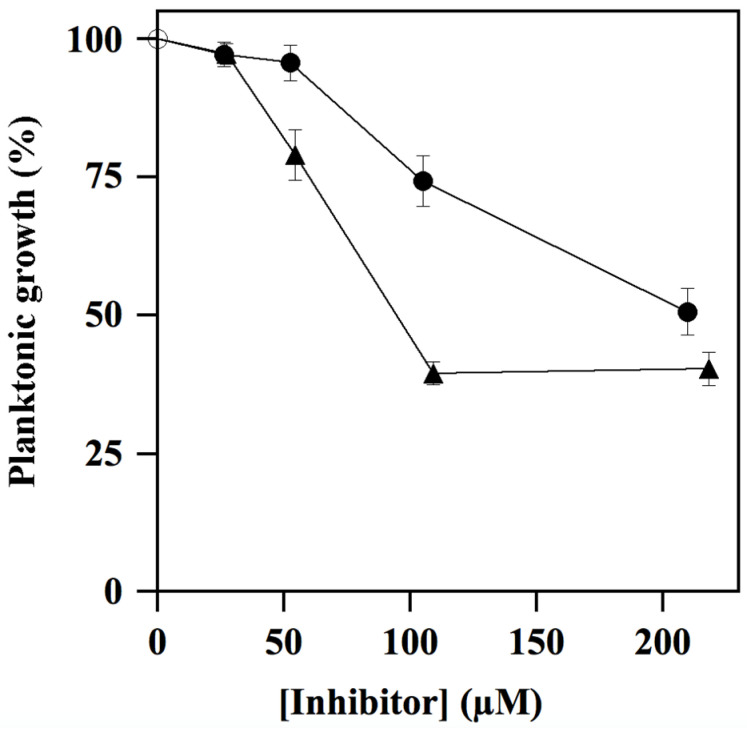
Effect of **ALS-31** and **ALS-31-9** on the planktonic growth of *Streptococcus mutans* cultures. The antimicrobial activity of *S. mutans* was investigated and evaluated, as indicated in Materials and Methods, in the absence (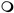) or in the presence of the indicated concentration of **ALS-31** (●) or **ALS-31-9** (▲).

The antimicrobial activity towards *S. mutans* cultures by compounds **ALS-31** and **ALS-31-9** was assayed by broth microdilution method. As shown in Figure 8, the planktonic growth of *S. mutans* was inhibited by compound **ALS-31** and **ALS-31-9** in a dose-dependent way. In particular, the percentage of growth was reduced to 74.3 ± 4.5 or 50.7 ± 4.2 in the presence of 104 or 209 µM **ALS-31**, respectively. The analogue **ALS-31-9** is more potent than **ALS-31**, because the percentage of planktonic growth of *S. mutans* was reduced to 79.0 ± 4.6 or 39.5 ± 2.1 in the presence of lower **ALS-31-9** doses, i.e., 54 or 108 µM, respectively; no further reduction in growth was observed when the concentration of this analogue was 216 µM. These data suggest that **ALS-31** and **ALS-31-9** do not display an antimicrobial activity in *S. mutans* suspensions up to the respective concentrations of 50 µM or 25 µM.

**Figure 9:**
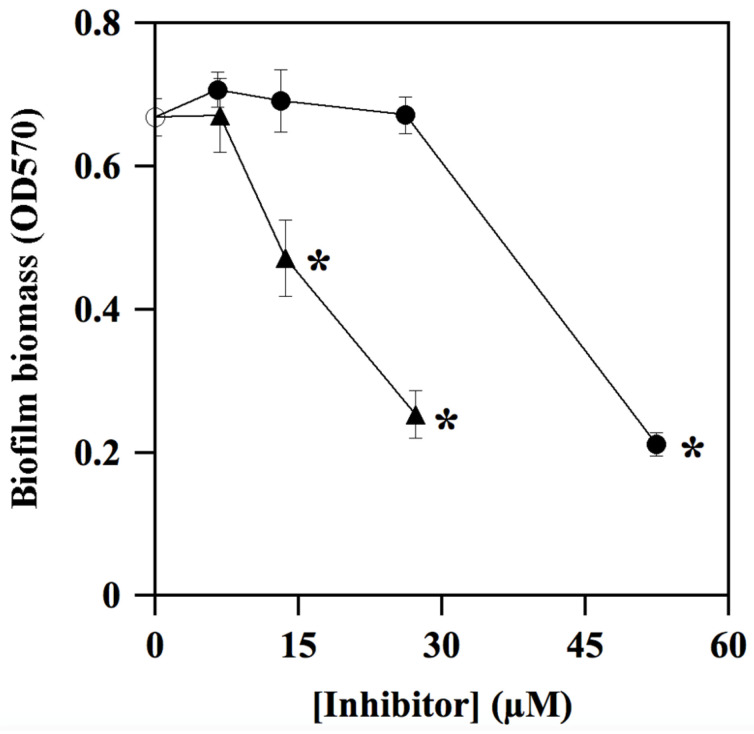
Effect of **ALS-31** and **ALS-31-9** on the antibiofilm activity of *Streptococcus mutans* cultures. The inhibition of total biofilm biomass formation of *S. mutans* was investigated and evaluated, as indicated in Materials and Methods, in the absence (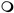) or in the presence of the indicated concentration of **ALS-31** (●) or **ALS-31-9** (▲). (**∗**): *p* < 0.0001 compared to control.

During its colonization of the dental surface, *S. mutans* is capable of forming thin biofilms on teeth, thus giving rise to dental plaque and caries. For this reason, the antibiofilm activity of **ALS-31** and **ALS-31-9** in *S. mutans* cultures was investigated using the CV staining method. However, in order to exclude effects due to inhibition of the planktonic growth, the maximum inhibitor concentration chosen in this assay is 52.5 µM for **ALS-31** and 27.3 µM for **ALS-31-9**. As shown in Figure 9, both substances are capable of inhibiting the total biofilm biomass formation in *S. mutans* cultures, although with a different potency. Indeed, when **ALS-31** is present at 52.5 µM, the formation of the biofilm is greatly reduced, accounting for 68.2% inhibition; no effects are observed with serial dilutions from this **ALS-31** concentration. On the other hand, the analogue **ALS-31-9** displays greater inhibition potency, as emerging from the values of 62.1% or 29.4% measured at 27.3 µM or 13.5 µM, respectively. Therefore, **ALS-31** and, even more, **ALS-31-9** could be considered as promising compounds for the design of molecules capable of inhibiting the formation of the thin biofilm on teeth during the colonization of the oral cavity by *S. mutans*.

## 4. Conclusions

*Streptococcus mutans* is a pathogenic facultative microaerophile playing an important role in the development of dental caries. *S. mutans* is capable of growing either in aerobic or anaerobic conditions, even though its energetic metabolism is essentially anaerobic. The leading role against the damages caused by reactive oxygen species, eventually formed during an aerobic stress, is sustained by the cambialistic *Sm*SOD. During colonization of the oral cavity by *S. mutans* since the appearance of the first deciduous teeth, it is likely that the activity of *Sm*SOD, an enzyme greatly regulated by which metal cation (Fe or Mn) is present in its active site, is essential for the survival of the pathogen within the biofilms covering teeth. These findings suggested that *Sm*SOD could be a convenient target for the identification of novel inhibitors of the enzyme functions, through a virtual screening study using two different approaches. In the list of 36 identified small molecules, possibly acting as metal chelators or disruptors of the active homodimeric interface of *Sm*SOD, **ALS-31** possessed the desired properties. Indeed, this compound was capable of inhibiting the activity of purified *Sm*SOD, as well as the planktonic growth and the biofilm formation of *S. mutans*. Its mechanism of action was investigated, and gel-filtration experiments clearly showed that **ALS-31** caused the dissociation of the active homodimer of *Sm*SOD in two monomers, a finding confirmed by the predicted binding mode of the inhibitor at the dimer interface of the enzyme. A preliminary lead optimization program was realised and the derivative **ALS-31-9** possessed a greater inhibition potency compared to that displayed by the lead compound. Our results could disclose novel strategies for the fight against dental caries, based on the identification of molecules acting on a crucial antioxidant enzyme target of *S. mutans*. Concerning the possible application of the identified novel molecules for treatment of dental caries, we can envisage their usage as a local topical medication, as the compounds have a direct antibiofilm activity on dental plaque formation by *S. mutans*.

## Figures and Tables

**Table 1:** Residual activity of *Sm*SOD in the presence of compounds selected by virtual screening.

Compound	Residual SOD Activity (%) in the Presence of [Compound] ^(*a*)^		
	20 µM	50 µM	100 µM
**ALS-2**	110	93	106
**ALS-3**	109	124	95
**ALS-4**	109	106	126
**ALS-5**	110	104	122
**ALS-6**	107	101	122
**ALS-7**	115	105	144
**ALS-9**	104	101	116
**ALS-10**	130	101	115
**ALS-11**	114	104	131
**ALS-12**	80	90	104
**ALS-13**	77	83	97
**ALS-14**	90	88	115
**ALS-16**	85	93	133
**ALS-17**	89	87	126
**ALS-18**	94	95	98
**ALS-19**	99	95	91
**ALS-20**	88	88	87
**ALS-21**	95	87	90
**ALS-22**	89	90	97
**ALS-23**	98	106	99
**ALS-24**	96	80	110
**ALS-25**	102	83	107
**ALS-26**	100	71	99
**ALS-27**	102	79	98
**ALS-29**	99	86	92
**ALS-30**	114	81	127
**ALS-31**	92	71	56
**ALS-32**	101	87	136
**ALS-33**	108	73	97
**ALS-34**	111	79	120
**ALS-35**	99	85	119
**ALS-36**	113	76	117

^(*a*)^ Activity of *Sm*SOD in the absence of compound equal to 100.

**Table 2:** Structure of **ALS-31** derivatives and their effect on *Sm*SOD activity.

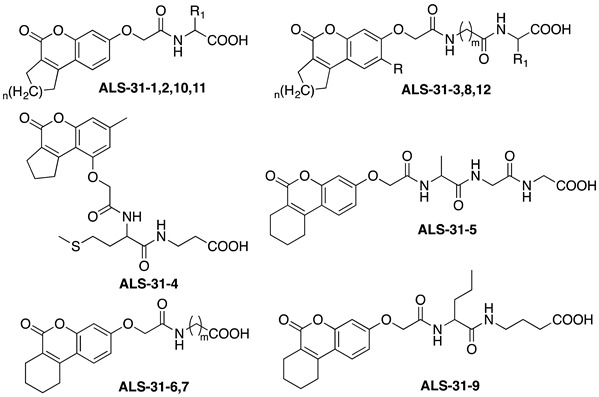								
**Code**	**ID**	**n**	**m**	**R**	**R_1_**	**Residual SOD Activity (%) in the Presence of [Compound] ^(*a*)^**		**IC_50_** **(µM) ^(*b*)^**
						**50 µM**	**100 µM**	
1098459	**ALS-31-1**	1	-	-	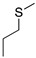	104.1	97.2	n.d.
1098464	**ALS-31-2**	1	-	-	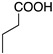	89.8	113.1	n.d.
1098679	**ALS-31-3**	1	2	H	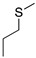	78.8	103.6	n.d.
1098687	**ALS-31-4**	-	-	-	-	92.6	96.8	n.d.
1098708	**ALS-31-5**	-	-	-	-	93.4	99.6	n.d.
1097658	**ALS-31-6**	-	3	-	-	75.1	78.0	127 ± 12
1097659	**ALS-31-7**	-	2	-	-	95.4	125.0	n.d.
1098702	**ALS-31-8**	2	2	CH_3_	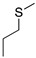	69.2	76.9	292 ± 30
1099294	**ALS-31-9**	-	-	-	-	65.3	50.3	64 ± 15
6235929	**ALS-31-10**	2	-	-	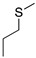	75.0	68.9	139 ± 8
1098529	**ALS-31-11**	2	-	-	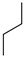	83.7	125.1	n.d.
1098688	**ALS-31-12**	2	1	H	H	75.6	125.3	n.d.

^(*a*)^ Activity of *Sm*SOD in the absence of compound equal to 100. ^(*b*)^ Not determined.

## Data Availability

Data are contained within the article and Supplementary Materials.

## Supplementary Materials

The following supporting information can be downloaded at: https://www.mdpi.com/article/10.3390/antiox11040785/s1. Figure S1: Sequence alignments of mature forms of *Sm*SOD, *St*SOD, Fe-SOD from *E. coli* and Mn–SOD from rat mitochondria; Table S1: Compounds identified by virtual screening; Table S2. Values of IC_50_ obtained for various SODs in the presence of **ALS-31**.
