# Supplementary material for: In Silico Identification of Novel Inhibitors Targeting the Homodimeric Interface of Superoxide Dismutase from the Dental Pathogen Streptococcus mutans
Source: Antioxidants (Basel). 2022 Apr 15;11(4):785. doi: 10.3390/antiox11040785 (PMC9029323; doi:10.3390/antiox11040785)
Supplement: Supplementary file 1 [file antioxidants-11-00785-s001.zip › antioxidants-1662076-supplementary.pdf]

Supporting Information for

In silico identification of novel inhibitors targeting the homodimeric interface of superoxide dismutase from the dental pathogen *Streptococcus mutans*

Carmen Cerchia [1,†], Emanuela Roscetto [2,†], Rosarita Nasso [2,3], Maria Rosaria Catania [2], Emmanuele De Vendittis [2], Antonio Lavecchia [1], Mariorosario Masullo [2,3,4,*] and Rosario Rullo [2,5,*]

[1] Department of Pharmacy, "Drug Discovery" Laboratory, University of Naples Federico II, 80131 Naples, Italy; carmen.cerchia@unina.it (C.C.); antonio.lavecchia@unina.it (A.L.)

[2] Department of Molecular Medicine and Medical Biotechnology, University of Naples Federico II, 80131 Naples, Italy; emanuela.roscetto@unina.it (E.R.); mariarosaria.catania@unina.it (M.R.C.); devendit@unina.it (E.D.V.)

[3] Department of Human Movement Sciences and Wellness, University of Naples "Parthenope", 80133 Naples, Italy; rosarita.nasso@collaboratore.uniparthenope.it (R.N.); mario.masullo@uniparthenope.it (M.M.)

[4] CEINGE, Biotecnologie Avanzate S.C.a R.L., 80145 Naples, Italy

[5] Institute for the Animal Production Systems in the Mediterranean Environment, 80147 Naples, Italy; rosario.rullo@cnr.it (R.R.)

Table of Contents

Figure S1. Sequence alignments of mature forms of *Sm*SOD, *St*SOD, Fe-SOD from *E. coli* and Mn–SOD from rat mitochondria
Table S1. Compounds identified by virtual screening
Table S2. Values of IC_{50} obtained for various SODs in the presence of **ALS-31**

```
SmSOD             AILLPDLPYA YDALEPYIDA ETMTLHHDKH HATYVANANA ALEKHPEIGE NLEVLLADVE   60
StSOD             AIILPDLPYA YDALEPYIDA ETMTLHHDKH HATYVANANA ALEKHPEIGE DLEALLADVE
Fe-SOD (E.coli)   SFELPALPYA KDALAPHISA ETIEYHYGKH HQTYVTNLNN LIKGTAFEGK SLEEIIRSSE
Mn-SOD (rat)      KHSLPDLPYD YGALEPHINA QIMQLHHSKH HATYVNNLNV TEEKYH---- --EALAKGDV
                     ** ***   .** *:*.* : :  *:.** * *** * *    :          * :  .

SmSOD             QIPADIRQSL INNGGGHLNH ALFWELLSPE KT-KVTAEVA AAINEAFGSF DDFKAAFTAA  119
StSOD             KIPADIRQAL INNGGGHLNH ALFWELLSPE KQ-EPTAEVA AAINEAFGSF EAFQEVFTTS
Fe-SOD (E.coli)   G-------GV FNNAAQVWNH TFYWNCLAPN AGGEPTGKVA EAIAASFGSF ADFKAQFTDA
Mn-SOD (rat)      TTQVALQPAL KFNGGGHINH SIFWTNLSPK GGGEPKGELL EAIKRDFGSF EKFKEKLTAV
                          .:   *..   ** :::*  *:*:    : ..::   **   ****   *:  :*

SmSOD             ATTRFGSGWA WLVVDKE-GK LEVTSTANQD TPI--SQGLK PILALDVWEH AYYLNYRNVR  176
StSOD             ATTRFGSGWA WLVVNAE-GK LEVVSTPNQD TPI--SDGKK PILALDVWEH AYYLKYRNVR
Fe-SOD (E.coli)   AIKNFGSGWT WLVKNSD-GK LAIVSTSNAG TPL--TTDAT PLLTVDVWEH AYYIDYRNAR
Mn-SOD (rat)      SVGVQGSGWG WLGFNKEQGR LQIAACSNQD -PLQGTTGLI PLLGIDVWEH AYYLQYKNVR
                  :    ****  **  : : *. * :.: .* .  *:  : .   *:* :***** ***:.*.*.*


SmSOD             PNYIKAFFEV INWNTVARLY AEALTK  202
StSOD             PNYIKAFFEI INWNKVAELY AEA--K
Fe-SOD (E.coli)   PGYLEHFWAL VNWEFVAKNL AA----
Mn-SOD (rat)      PDYLKAIWNV INWENVSQRY IVC-KK
                  *.*:: :: : :**: *:
```

Figure S1. Sequence alignments of mature forms of *Sm*SOD, *St*SOD, Fe-SOD from *E. coli* and ~~the~~ Mn–SOD from rat mitochondria. Alignment was performed with MUSCLE [Edgar, R.C. MUSCLE: Multiple sequence alignment with high accuracy and high throughput. *Nucleic Acids Res.* **2004**, *32*, 1792–1797]. The residues mainly involved in dimer assembly (E21, H30, S126, E165, H166, Y169, R173, residue numbering referred to the PDB structure 4YIP) are highlighted in pink.

Table S1. Compounds identified by virtual screening.

N.	Code	Approach	ID	Structure
**1**	1149684	LBVS	ALS-1	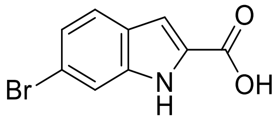
**2**	1088043	LBVS	ALS-2	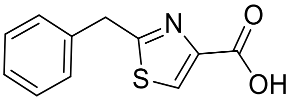
**3**	1794806	LBVS	ALS-3	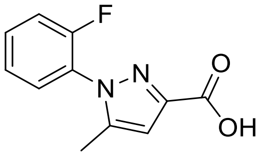
**4**	2251077	LBVS	ALS-4	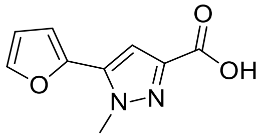
**5**	1593018	LBVS	ALS-5	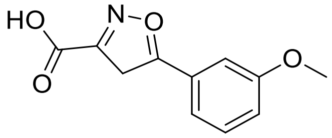
**6**	2201826	LBVS	ALS-6	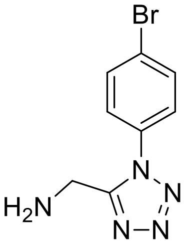
**7**	3543934	LBVS	ALS-7	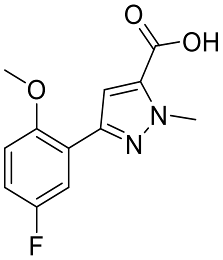
**8**	5042299	LBVS	ALS-8	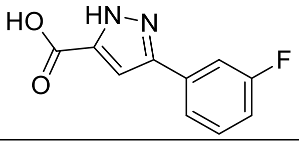
**9**	1973472	LBVS	ALS-9	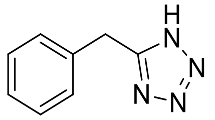
**10**	2229874	LBVS	ALS-10	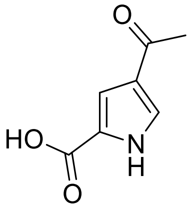

**11**	9080680	LBVS	ALS-11	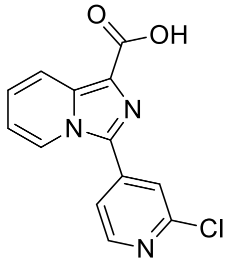
**12**	4041753	LBVS	ALS-12	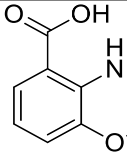
**13**	1780792	LBVS	ALS-13	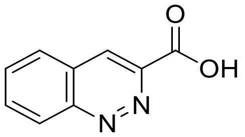
**14**	6328802	LBVS	ALS-14	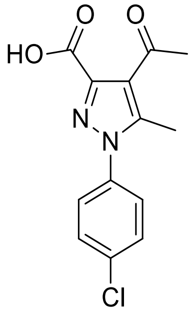
**15**	7119870050	LBVS	ALS-15	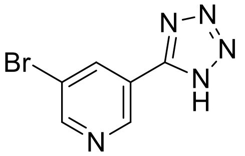
**16**	3339531	LBVS	ALS-16	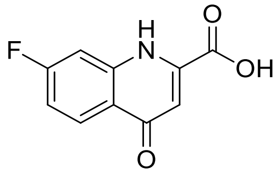
**17**	1975653	LBVS	ALS-17	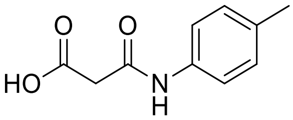
**18**	0128660069	SBVS	ALS-18	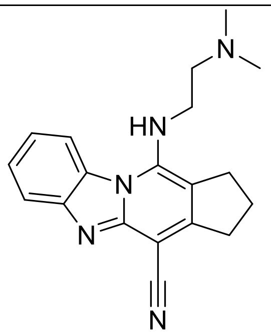
**19**	1086703	LBVS	ALS-19	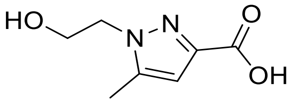

**20**	1045880	LBVS	ALS-20	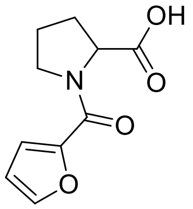
**21**	1331803	SBVS	ALS-21	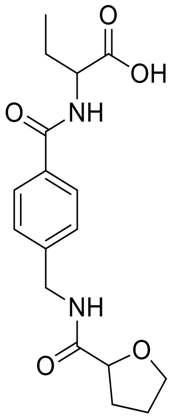
**22**	6923424	LBVS	ALS-22	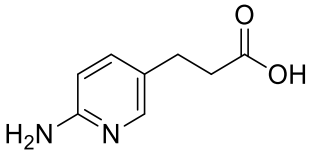
**23**	6051822	LBVS	ALS-23	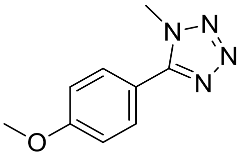
**24**	7138831	LBVS	ALS-24	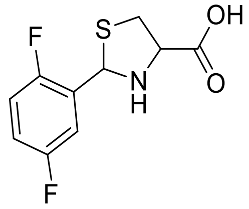
**25**	2222682	LBVS	ALS-25	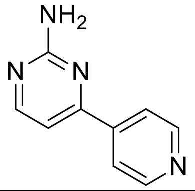
**26**	2214908	LBVS	ALS-26	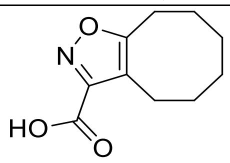
**27**	2270454	LBVS	ALS-27	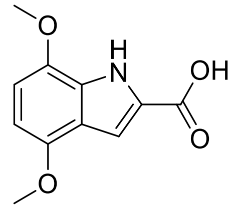
**28**	3533961	LBVS	ALS-28	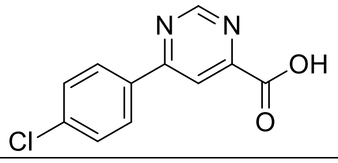

**29**	2195404	SBVS	ALS-29	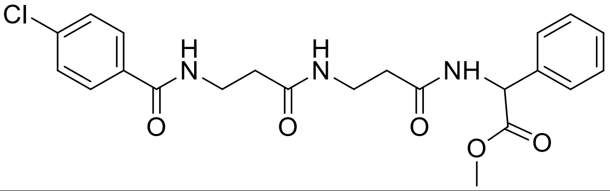
**30**	4794984	SBVS	ALS-30	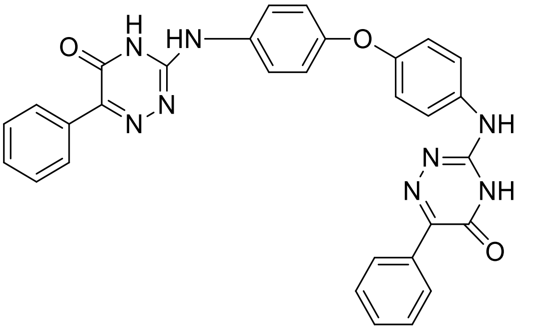
**31**	1098697	SBVS	ALS-31	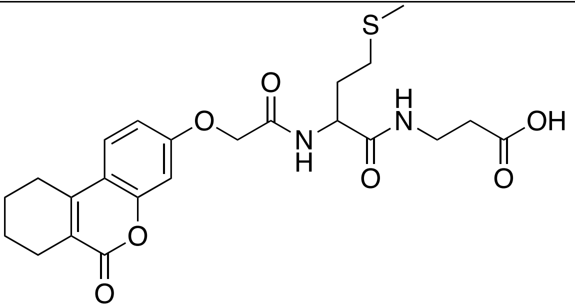
**32**	3460720	SBVS	ALS-32	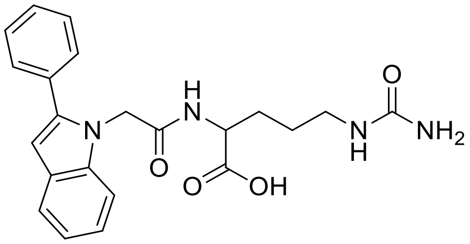
**33**	6242859	SBVS	ALS-33	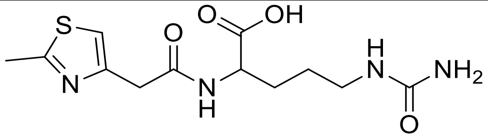
**34**	6244314	SBVS	ALS-34	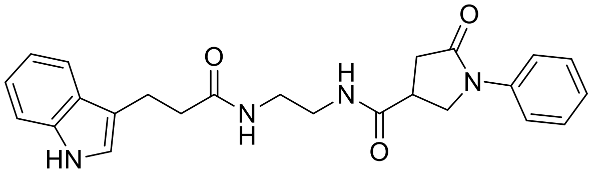
**35**	7131627	SBVS	ALS-35	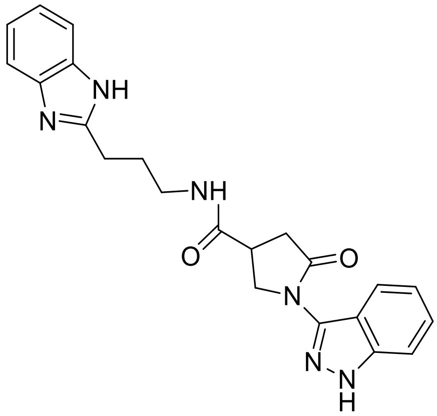

**36**	7567692	SBVS	ALS-36	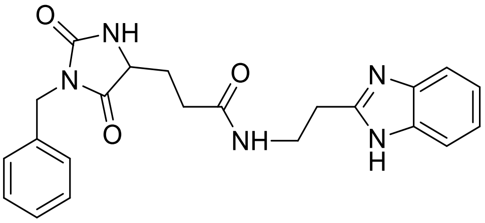

Table S2. Values of IC_{50} obtained for various SODs in the presence of **ALS-31**

SOD group	Source	IC_{50} (μM)
Cambialistic	*Streptococcus mutans*	159 ± 19
"	*Streptococcus thermophilus*	218 ± 50
Fe-SOD	*Escherichia coli*	123 ± 20
Mn-SOD	Rat mitochondria	161 ± 56
Cu/Zn-SOD	Bovine erythrocytes	146 ± 25
